# Navigating the AI Innovation: A Publisher's Balancing Act with Fundamental Principles

**DOI:** 10.17159/2078-516X/2024/v36i1a17574

**Published:** 2024-02-15

**Authors:** Mike Lambert

**Affiliations:** Editor-in-chief

Since the editorial in January 2023,^[1]^ the South African Journal of Sports Medicine (SAJSM) has successfully transitioned to a new publishing model that includes author publication costs. The goal of this decision was to make the journal self-sustaining and reduce the financial burden on the South African Sports Medicine Association, which has supported the journal for decades. Forty-eight papers were submitted for review in 2023. This was a slight decrease from the previous year, possibly due to the new publication charges. Fifty per cent of these papers were accepted for publication. The SAJSM also published a series of papers for the Special Cricket Edition. These papers were popular, with a combined total of 3769 abstract reads in 2023.

The paper with the most downloads in 2023 was “*The effectiveness of intratissue percutaneous electrolysis for the treatment of tendinopathy: a systematic review*”^[2]^ which was downloaded 767 times, followed by “*Management of lumbar bone stress injury in cricket fast bowlers and other athletes*”^[3]^ (n = 489 downloads).

According to the Scimago Journal and Country Rank (SJR) the SAJSM has a Q3 ranking, which means the journal is in the 50–75% range compared to other journals in the same subject category. A Q3 ranking is a moderate ranking. The SAJSM rating can be improved by increasing the quantity and quality of papers submitted to the journal.

iThenticate screens all papers submitted to the SAJSM before they are sent for review. Three papers were rejected in 2023 before being sent for review as they had an unacceptably high level of similarity with previously published work. The editorial team is vigilant against plagiarism as authors attempt to take shortcuts by copying other works without proper citation.

Finding quality reviewers remains a challenge. Five training courses were held in 2023 in which thirty reviewers were trained. The success of these courses will depend on the number of attendees accepting invitations to review as the year progresses. Surprisingly few reviewers submitted their details to the Web of Science after they completed their review. There will be a drive to increase the number who do so in 2024. Submitting details to the Web of Science is important for reviewers as it provides a valuable opportunity for recognition and professional development. While reviewing is often anonymous with no tangible rewards, having one's reviews documented in the Web of Science database allows reviewers to enhance their academic CVs.

The editorial's theme last year was artificial intelligence's impact on the publishing industry.^[1]^ The take-home message for journals and publishers was that the “challenge is to remain alert for positive and negative effects and adjust accordingly”. This theme remains pertinent in 2024. For example, ChatGPT can assist researchers and authors in quickly generating content. This is particularly useful for drafting initial ideas or creating outlines. ChatGPT may expedite the gathering of relevant information in preparation for a more formal literature review. ChatGPT's language capabilities can be harnessed for efficient translation of scientific content. ChatGPT can also be used as a tool for brainstorming and generating innovative ideas and as a creative writing assistant, aiding in the initial stages of research projects. These positive features are challenged by drawbacks. For example, researchers might overly rely on ChatGPT for writing tasks, leading to a shortcut culture. This could hinder the development of essential skills in scientific research. This problem will not manifest immediately. It may be analogous to an athlete relying on drugs for short-term gains in performance, but in the long term, these substances can adversely affect the body, leading to injuries, organ damage, and a decline in overall well-being. Similarly, a researcher may benefit in the short term because of gains in productivity as the tool can assist in rapidly generating content, and in quickly completing writing tasks. However, overdependence on the tool could hinder the development of essential academic skills, such as critical thinking, originality, and the ability to formulate ideas independently. Scientific inquiry and problem-solving require these skills. Without them, career progression is limited.

Besides ChatGPT, several AI-powered tools can benefit authors and researchers in various stages of the research and writing process. For instance, Grammarly serves as a writing assistant, improving grammar, spelling, and clarity while providing real-time suggestions for correcting errors. Scite is an AI tool that helps researchers evaluate the reliability of scientific articles by analysing and classifying citation statements. Manuscript Matcher, aligned with the reference manager EndNote, uses AI to recommend potential journals for authors to submit their research papers. These are a few of the many examples.

From a content perspective, the journal can expect more papers on sport-related concussions as this topic remains high on the list of research priorities. Also, research is expected to track the trends in health and fitness. For example, in 2024, wearable technology is expected to continue its reign as the top fitness trend, a position it has held consistently since 2016. This indicates a sustained consumer interest in tracking health data.^[4]^ The fitness industry is also seeing a resurgence of in-person activities, with boutique fitness studios and subscription-based memberships gaining popularity as the world adapts to a post-pandemic normal. Additionally, there is a growing emphasis on employing certified fitness professionals, reflecting a trend towards professionalisation and quality in fitness services.^[4]^ A newcomer to the list of trends is Data-Driven Training Technology. This is linked to the popularity of wearable technology, where clients can use real-time data output, such as heart rate, velocity, and speed, to guide their training sessions.^[4]^

As publishers navigating the evolving AI landscape, we strive to strike a harmonious balance between innovation and robust principles, ensuring our work contributes meaningfully to the collective knowledge pool. SAJSM eagerly embraces 2024, ready to face the changing landscape of sports and exercise medicine!
